# Peptidoglycan from *Bacillus anthracis* Inhibits Human Macrophage Efferocytosis in Part by Reducing Cell Surface Expression of MERTK and TIM-3

**DOI:** 10.4049/immunohorizons.2300109

**Published:** 2024-03-22

**Authors:** Joshua S. Mytych, Zijian Pan, Charmaine Lopez-Davis, Nancy Redinger, Christina Lawrence, Jadith Ziegler, Narcis I. Popescu, Judith A. James, A. Darise Farris

**Affiliations:** * Arthritis and Clinical Immunology Program, Oklahoma Medical Research Foundation, Oklahoma City, OK; † Department of Microbiology and Immunology, University of Oklahoma Health Sciences Center, Oklahoma City, OK

## Abstract

*Bacillus anthracis* peptidoglycan (PGN) is a major component of the bacterial cell wall and a key pathogen-associated molecular pattern contributing to anthrax pathology, including organ dysfunction and coagulopathy. Increases in apoptotic leukocytes are a late-stage feature of anthrax and sepsis, suggesting there is a defect in apoptotic clearance. In this study, we tested the hypothesis that *B. anthracis* PGN inhibits the capacity of human monocyte-derived macrophages (MΦ) to efferocytose apoptotic cells. Exposure of CD163^+^CD206^+^ MΦ to PGN for 24 h impaired efferocytosis in a manner dependent on human serum opsonins but independent of complement component C3. PGN treatment reduced cell surface expression of the proefferocytic signaling receptors MERTK, TYRO3, AXL, integrin α_V_β_5_, CD36, and TIM-3, whereas TIM-1, α_V_β_3_, CD300b, CD300f, STABILIN-1, and STABILIN-2 were unaffected. ADAM17 is a major membrane-bound protease implicated in mediating efferocytotic receptor cleavage. We found multiple ADAM17-mediated substrates increased in PGN-treated supernatant, suggesting involvement of membrane-bound proteases. ADAM17 inhibitors TAPI-0 and Marimastat prevented TNF release, indicating effective protease inhibition, and modestly increased cell-surface levels of MerTK and TIM-3 but only partially restored efferocytic capacity by PGN-treated MΦ. We conclude that human serum factors are required for optimal recognition of PGN by human MΦ and that *B. anthracis* PGN inhibits efferocytosis in part by reducing cell surface expression of MERTK and TIM-3.

## Introduction

*Bacillus anthracis*, the causative agent of anthrax infection, is a large, spore-forming, and toxin-producing Gram-positive organism that typically infects herbivores. Humans can be infected through various routes, including inhalation, ingestion, or cutaneous exposure to spores, the most severe being inhalation, which can lead to a rapid and highly fatal infection (1). Latent spores migrate to and germinate in the mediastinal lymph nodes, followed by progression to fulminant infection characterized by high levels of circulating bacteria and sepsis-like features (2). Bacterial sepsis is a leading cause of mortality worldwide and has limited treatment options (3). Accumulation of uncleared apoptotic leukocytes is a known hallmark of sepsis (4) and is accompanied by increased levels of circulating nucleosomes (5). Circulating nucleosomes can be released from uncleared apoptotic cells that progress to secondary necrosis (6), promoting micro- and macrovascular leakage (7) and contributing to organ dysfunction (8).

Macrophages (MФ), either tissue resident or those present in secondary lymphoid organs, are responsible for the clearance of apoptotic cells through a process termed “efferocytosis” (9–11). Highly efferocytic MΦ tend to have an M2-like, or alternative activation, program (12, 13), expressing CD163 and CD206 (14), and acquire anti-inflammatory properties that actively dampen inflammation by secreting proresolving mediators (15). Highly efferocytic MΦ can be modeled in vitro by polarization with IL-10, IL-4, and/or dexamethasone (16, 17). Defects in efferocytosis amplify inflammation in sepsis by increasing the levels of circulating nucleosomes and other host damage-associated molecular patterns (DAMPs) (18). Recognition of apoptotic cells is a complex process involving tethering receptors that, on MФ, bind directly to phosphatidylserine on apoptotic cells or receptors that require soluble phosphatidylserine-binding bridge proteins (19). Currently, there are at least 12 well-established receptors that signal for apoptotic cell engulfment: TYRO3, AXL, MERTK, integrins α_V_β_3_ and α_V_β_5_, CD300b, CD300f, STABILIN-1 and -2, TIM-1 and -3, and BAI-1 (20). Of these, TYRO3, AXL, and MERTK require bridging by GAS6, protein S, or GAL-3, and integrins α_V_β_3_ and α_V_β_5_ require bridging via MFGE8 or DEL-1. The remaining seven receptors directly bind to apoptotic cells (20, 21). Efferocytic receptors are thought to be regulated at the cell surface by metalloproteases, and proteolysis of surface-exposed TYRO3, AXL, MERTK, TIM-1, and TIM-3 has been reported in mouse and human MФ (22). Both matrix metalloproteinase (MMP) and a disintegrin and metalloproteinase (ADAM) families are known to cleave efferocytosis receptors at the cell surface (23). Loss of these receptors has been shown to correlate with disease phenotypes and may be useful as biomarkers in multiple diseases, including lupus and Sjogren’s disease, where defects in efferocytosis are thought to contribute to disease progression (24). Of the metalloproteinases known to regulate efferocytosis, ADAM17 is a key sheddase responsible for cleaving more than 80 substrates, including TNF and MERTK, and has been implicated as a regulator of efferocytosis (25, 26).

We previously showed that *B. anthracis* edema toxin, an adenylate cyclase that induces supraphysiologic levels of cAMP, impairs efferocytosis through inhibition of Rac1 activation and altered phosphorylation of proteins involved in actin reorganization (27). In this study, we focus on peptidoglycan (PGN), a major component of the cell wall in Gram-positive bacteria and a significant factor in anthrax-mediated sepsis that contributes to vascular and organ damage and mortality in nonhuman primate models of anthrax sepsis (28, 29). Response to PGN was initially thought to be at the cell surface and TLR2 dependent (30); however, recent work has challenged this view and showed that impurities from lipoteichoic acid resulted in TLR2 activation (31). Further work elucidated that purified PGN preparations lacking lipoteichoic acid activate cytosolic nucleotide-binding oligomerization domain (NOD) receptors (32–34). Additional work using human monocytes, neutrophils, and dendritic cells suggests that polymeric PGN activates NOD in human immune cells more potently in the presence of human serum opsonins (35–37); however, this has yet to be shown for human MФ. In this study, we demonstrate that recognition of *B. anthracis* PGN by human MΦ is enhanced by human serum factors and that PGN inhibits human MΦ efferocytosis in part by reducing cell surface expression of MERTK and TIM-3.

## Materials and Methods

### Differentiation of monocytes to M2-like MФ

On day 0, human mononuclear cells were isolated from fresh buffy coats (Our Blood Institute, Oklahoma City, OK) using Lympholyte (Cedarlane Laboratories, Burlington, ON, Canada) according to the manufacturer’s protocol (see Supplemental Fig. 1 for timeline of assay). Following isolation, PBMCs were counted and plated on 100-mm petri dishes (Corning, Corning, NY , catalog no. 430591; 100-mm × 20-mm nontreated tissue culture dishes) at 4 × 10^6^ cells/ml in 25 ml per dish in IMDM with glutamine (Thermo Fisher Scientific, Waltham, MA, catalog no. 12440053) with no other additives. Cells were allowed to adhere for 1 h at 37°C in 5% CO_2_, followed by gentle removal of media and replacement with 20 ml/dish of complete medium (IMDM, 10% heat-inactivated FBS [hiFBS], 5 mM penicillin-streptomycin antibiotic) supplemented with 50 ng/ml M-CSF (PeproTech, Rocky Hill, NJ, catalog no. 300-25). On days 2 and 5, half of the media was exchanged with fresh media containing 100 ng/ml M-CSF to replenish spent M-CSF (final M-CSF concentration was 50 ng/ml). On day 6, MΦ were polarized to a tissue-like/alternative phenotype by adding 500 nM dexamethasone and culturing for 24 h (Sigma-Aldrich, St. Louis, MO, catalog no. D4902). All polarized MΦ expressed CD206 and CD163, as we previously published (27).

### PGN isolation from *B. anthracis*

PGN was purified from the Sterne strain BA781 (Δlef243/Δcy244/Δpag242) obtained through the NIH Biodefense and Emerging Infections Research Resources Repository (National Institute of Allergy and Infectious Diseases). This strain is a triple-deletion mutant lacking anthrax toxins and the pXO2 plasmid responsible for capsule production. Methodologies for purification and analytical characterization of PGN have been described in detail previously (33). Briefly, stationary phase cultures were pelleted by centrifugation, then boiled in 8% SDS to remove phospholipids and lipoproteins, followed by sequential digestions with nucleases and proteinase K. Secondary cell wall polysaccharides and teichoic acids were removed by cold hydrolysis with 48% (w/v) hydrofluoric acid (38). Purified PGN was extensively washed with endotoxin-free water, sonicated to break up large fragments, aliquoted, and dried by centrifugal vacuum evaporation. Amino acid composition of the polypeptide bridge was assessed by HPLC after acid hydrolysis as previously described (33) and confirmed by mass spectrometry on select lots. The purified PGN contained only alanine, glutamate, and meso-diaminopimelic acid, consistent with the canonical structure for *B. anthracis* PGN (39). The purified PGN used in this study had a median particle size of 250 nm as measured by nanoscale cytometry (40) and was devoid of TLR agonists as evidenced by the lack of activation of TLR2 and TLR4 reporter cell lines (InvivoGen, San Diego, CA). Throughout the study, PGN was tested at 10 µg/ml, a concentration approximating the quantity of PGN that is released from a clinically relevant level of bacteria, 1 × 10^7^cfu/ml of heat-killed *B. anthracis* (41).

### PGN treatment

On day 7, 24 h after polarization with dexamethasone, MΦ were replated to 12-well Nunc UpCell plates (Thermo Fisher, catalog no. 150200). Briefly, 3 ml trypsin/EDTA solution (Sigma-Aldrich, catalog no. T4049-100) was added to each petri dish and incubated at 37°C in 5% CO_2_ for 10 min to detach cells. After detachment, 10 ml complete medium (containing 10% FBS) was added to neutralize trypsin activity. MФ were pelleted at 1200 rpm for 5 min, counted, and replated to the UpCell plates at 0.5 × 10^6^ cells/ml. After replating, MΦ were incubated for 16 or 24 h with 10 µg/ml highly purified *B. anthracis* PGN (33) (the same isolation batch was used for all experiments) in media supplemented with 50 ng/ml M-CSF and various serum additives as follows: 10% hiFBS, 10% heat-inactivated human serum (hihuS), 10% non-hihuS, or 10% non-hihuS + 200 µg/ml compstatin. Human serum used for all conditions was from the same lot of pooled human AB serum (Fisher Scientific, catalog no. BP2525-100). For heat inactivation, serum was placed in a 56°C water bath for 30 min and stored at −20°C until use. Compstatin was purchased from Selleckchemical (Houston, TX, catalog no. S8522). For ADAM17 inhibition, MΦ were treated with 10 µg/ml PGN and concomitantly with 30 µM TAPI-0 or Marimastat (Tocris, McKinley Place, MN, catalog nos. 5523, 2631).

### Preparation of apoptotic neutrophils

On the morning of day 8, human polymorphonuclear neutrophils (PMNs) were isolated by immunomagnetic negative selection from fresh healthy donor blood (EasySep Direct Human Neutrophil isolation kit, STEMCELL Technologies, catalog no. 19666). After isolation and counting, PMNs were resuspended to 5 × 10^6^ cells/ml in IMDM without additives, labeled with eFluor 670 according to the manufacturer’s protocol (Thermo Fisher, catalog no. 64-0840-90), then exposed to 217.8 mJ/cm^2^ UV irradiation for 3 min to induce apoptosis. Irradiated PMNs were then placed in a 37°C incubator with 5% CO_2_ for 4 h. After 4 h, an aliquot of PMNs was stained with annexin V and propidium iodide (Annexin-V Apoptosis Detection kit, eBioscience, catalog no. 88-8005-74) and assessed for the frequency of annexin V^+^/propidium iodide^−^ early apoptotic PMNs by flow cytometry. All apoptotic cell preparations contained ≥70% early apoptotic cells.

### Efferocytosis assay

On day 8, labeled apoptotic PMNs were coincubated with MΦ to assess efferocytosis. Prior to adding apoptotic PMNs, supernatants from cultured MФ were replaced with 0.5 ml/well of IMDM without serum. The collected supernatant was stored at −80°C until use for detection of secreted cytokines. After 4 h, irradiated eFluor 670–labeled PMNs were added to UpCell plate-containing MΦ at a 5:1 apoptotic PMN:MΦ ratio and incubated for 1 h (2.5 × 10^6^/ml PMN:0.5 × 10^6^/ml MΦ). After 1 h, UpCell plates were gently washed two times using 1 ml PBS per wash to remove unbound PMNs. UpCell plates were rested at room temperature for 10 min to allow MΦ detachment. To ensure complete removal, wells were washed once with 1 ml ice-cold PBS.

### Flow cytometry

MΦ (0.5 × 10^6^/tube) were stained for flow cytometry in 5-ml round-bottomed polystyrene tubes (Corning, catalog no. 352058). After pelleting the cells at 1200 rpm for 5 min, they were incubated for 10 min at room temperature with a combined Fc block solution (2 µl Human Fc block [BD Biosciences, San Jose, CA] + 3 µl type AB hihuS [Fisher Scientific, Waltham, MA, catalog no. BP2525-100] + 5 µl Brilliant Stain buffer [Thermo Fisher, Waltham, MA, catalog no. 00-4409-42]) per tube. Primary Abs were then added and, after gentle mixing, incubated for 25 min on ice. After washing the cells with FACS wash (2% FBS, 1 mM EDTA in PBS), unconjugated Abs were detected with secondary PE-conjugated Ab for 20 min on ice. After staining, all samples were washed with 2 ml FACS wash, fixed in 1% paraformaldehyde in 0.15 M NaCl, pH 7.4, and kept at 4° until analysis. Data were collected using an LSR II flow cytometer (BD Biosciences) and analyzed using FlowJo version 10.7.1 software (FlowJo LLC, Ashland, OR). The numbers of events recorded were 20,000/treatment for receptors and 40,000/treatment for efferocytosis assay. Abs and clones included in the efferocytosis mixture were from BioLegend (San Diego, CA) or from BD Biosciences: PE-conjugated anti-human CD11b (clone ICRF44), allophycocyanin cyanine 7–conjugated anti-human CD163 (clone GHI/61), PerCP-Cy5.5–conjugated anti-human CD206 (clone 15-2), Alexa Fluor 488–conjugated anti-human CD64 (clone 10.1) (BioLegend) BV421-conjugated anti-human CD66b (clone G10F5) (BD Biosciences). For detecting surface receptor expression, efferocytic receptors were all PE labeled and purchased from BioLegend CD36 (clone 5-271) or were purchased from R&D Systems as follows: DTK/TYRO3 (clone 96201), AXL (clone 108724), MERTK (clone 125518), α_V_β_3_ (clone 23C6), or α_V_β_5_ (clone P5H9), CD300f (clone UP-D2). Additional receptors were only available unconjugated and were purchased from R&D Systems: anti-human CD300b (polyclonal goat IgG, catalog no. AF2879) and stained with secondary Ab (PE-conjugated anti-goat IgG, catalog no. F0107). Anti-human STABILIN-1 and anti-human STABILIN-2 primary Abs (both polyclonal sheep IgG, catalog no. AF3825, catalog no. AF2645) were stained with a PE-conjugated secondary Ab (polyclonal anti-sheep IgG, catalog no. 5-001-A). Receptor expression was quantified using mean fluorescence intensity for each respective treatment group.

### Measurement of soluble analytes

Supernatants from PGN-treated MФ (24 h in non-hihuS) were tested for soluble analytes (AXL, CD36, CD44, ICAM-1, LOX-1, MERTK, RAGE, TIM-3, TNF, and TYRO3) using a custom Procarta Luminex assay from Thermo Fisher. Samples were diluted 1:5 or 1:10 in assay buffer and tested according to the manufacturer’s protocol. Results were read on a BioPlex 200 instrument and analyzed using BioPlex Manager software (Bio-Rad Laboratories, Hercules, CA).

### Data analysis

All data are presented as mean ± SEM, with the number of independent donors indicated in the figure legends. Results were analyzed by one-way or two-way ANOVA or a mixed-effect analysis followed by a Dunnett’s or Sidak’s multiple-comparison post hoc test (*p* < 0.05), a paired *t* test, or a nonlinear fit model as indicated in the figure legends. Graphs and statistics were generated in GraphPad Prism (GraphPad Prism version 9.2.0, GraphPad Software, La Jolla, CA).

## Results

### Human serum factors enhance human MФ recognition of *B. anthracis* PGN

**FIGURE 1.**
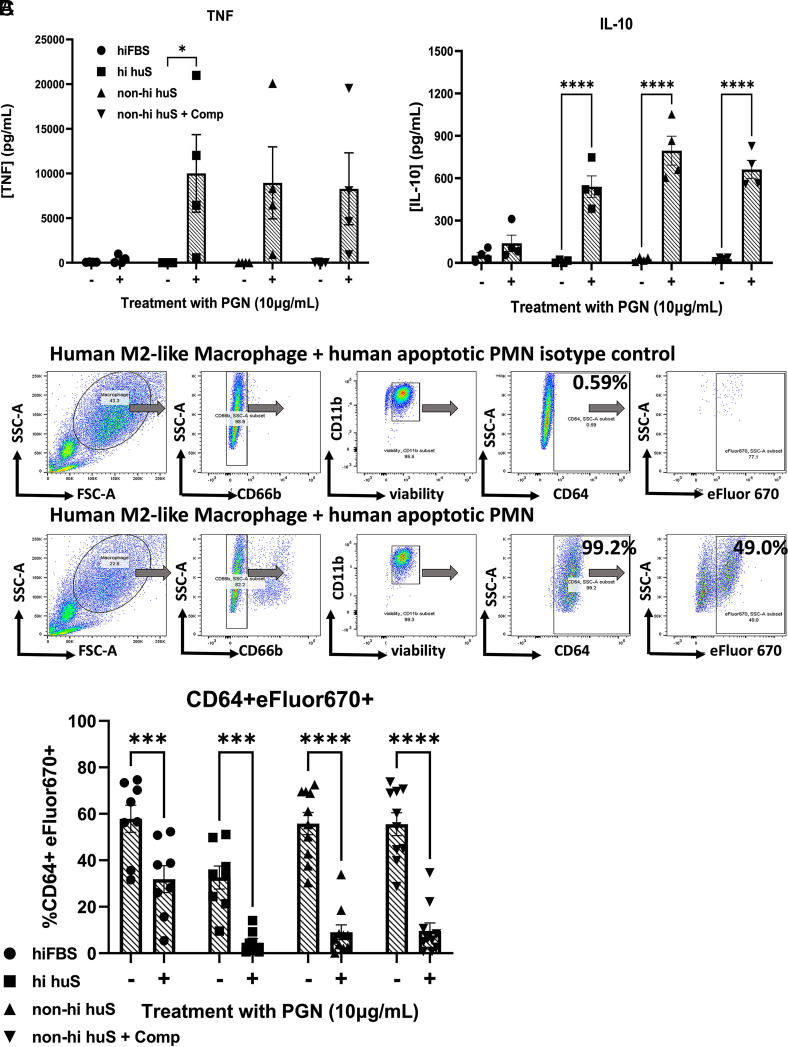
*B. anthracis* PGN inhibits efferocytosis in the presence of human serum. (**A**) Secreted TNF and IL-10 (24 h–treated MФ in various serum conditions) from four independent donors. (**B**) Gating strategy for flow cytometric evaluation of efferocytosis. Gating strategy: After gating on MΦ and excluding dead cells and debris using scatter properties (forward scatter [FSC], side scatter [SSC]), MΦ with CD66b^+^ surface-bound apoptotic PMNs are excluded. Next, live MΦ are gated as CD11b^+^ and Zombie Aqua viability dye negative. Finally, we gate on CD64^+^ MΦ and measure the percentage of intracellular apoptotic PMNs present (CD64^+^ eFluor 670^+^). (**C**) Percentage of CD64^+^ MФ containing apoptotic eFluor 670–labeled PMNs from the indicated serum conditions in the presence or absence of 10 μg/ml PGN from ≥5 independent donors analyzed by two-way ANOVA with Sidak’s multiple comparison test. *p < 0.05, ***p < 0.001, ****p < 0.0001.

To evaluate whether human serum opsonins are important for MΦ responses to PGN, we tested primary human MΦ for their capacity to produce cytokines in response to PGN in the presence of various serum conditions. We evaluated TNF and IL-10 production following exposure to 10 µg/ml purified *B. anthracis* PGN in the presence of the following:10% hiFBS, 10% hihuS, 10% non-heat-inactivated human serum (non-hihuS), and 10% non-hihuS pretreated with compstatin, a specific inhibitor of C3b-mediated opsonization (non-hihuS+Comp). We observed negligible production of TNF and IL-10 in PGN-treated MФ in the presence of hiFBS; however, in the presence of human serum, PGN induced increases in both TNF and IL-10 concentrations in supernatants (Fig. 1A). Of four donors tested, three showed an increase in both cytokines, whereas one donor showed notably low secretion of TNF, regardless of serum condition.

### *B. anthracis* PGN inhibits human MФ efferocytosis of apoptotic PMNs

Next, we tested whether exposure of M2-like MФ to PGN impacts their capacity to efferocytose human apoptotic neutrophils, also termed “polymorphonuclear leukocytes” (PMNs). MФ were pretreated with PGN for 24 h in the presence of the various serum conditions shown in Fig. 1A, then coincubated with primary apoptotic PMNs for 1 h, followed by assessment of efferocytosis using flow cytometry. Consistent with a human serum opsonin requirement for recognition of PGN by human MΦ, efferocytosis was modestly impaired in the presence of hiFBS but profoundly impaired in the presence of all human serum conditions (Fig. 1B, 1C). We also performed a dose–response assay to find the IC_50_ of PGN on efferocytosis using MΦ from three representative donors (Supplemental Fig. 2). PGN elicited similar responses in both non-hihuS and non-hihuS+Comp in its capacity to inhibit efferocytosis (IC_50_ = 1.46 µg/ml versus 1.49 µg/ml), reinforcing that complement component C3 contributes little to PGN’s inhibition of efferocytosis in our system.

### PGN treatment reduces cell surface expression of efferocytic receptors through proteolytic cleavage

**FIGURE 2.**
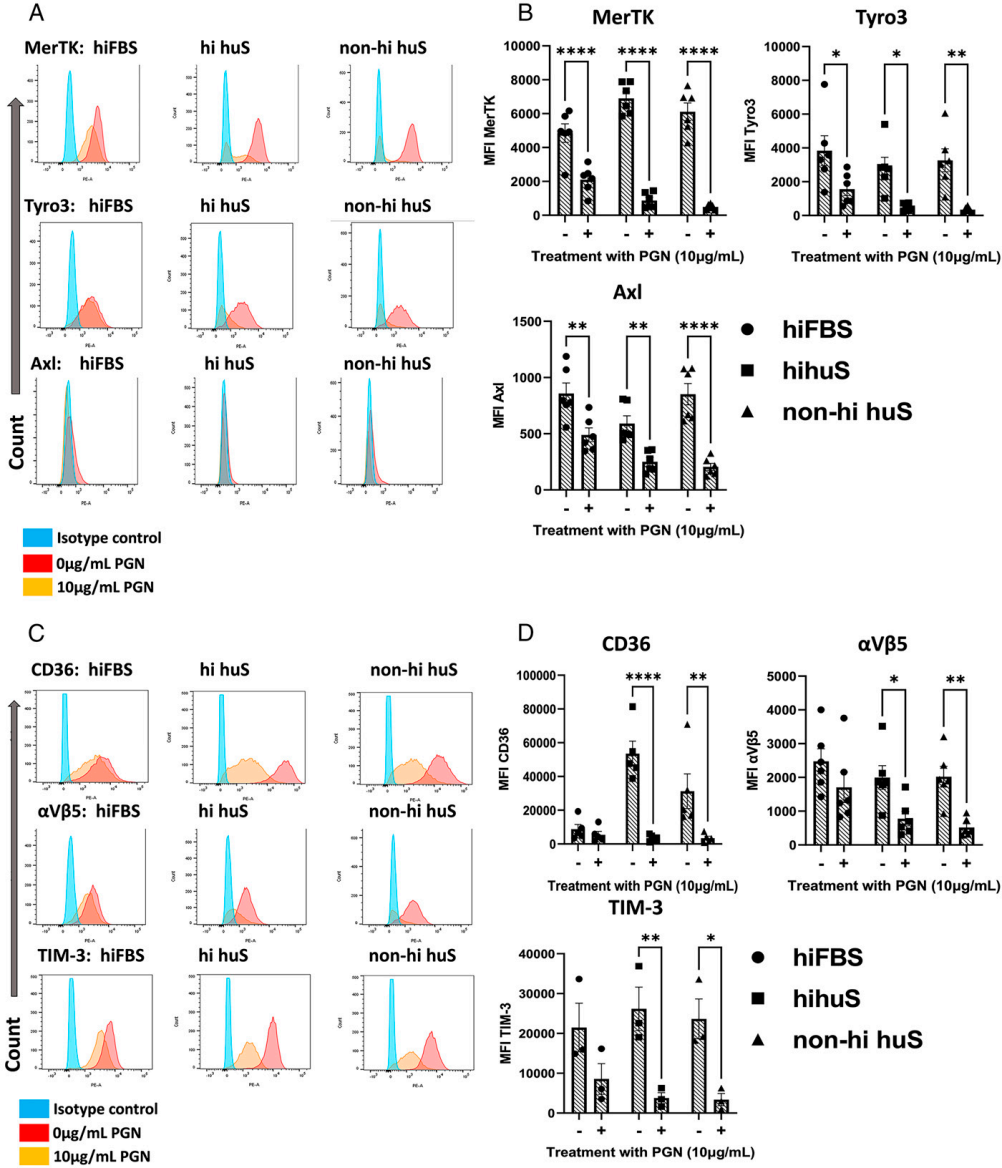
Human M2-like MΦ reduce efferocytic receptor expression in response to PGN. (**A** and **C**) Representative histograms of receptor expression. (**B** and **D**) Mean fluorescence intensity (MFI) of surface efferocytosis receptors from the indicated serum conditions from three or more independent donors analyzed by two-way ANOVA with Sidak’s multiple comparison test. *p < 0.05, **p < 0.01, ****p < 0.0001.

**FIGURE 3.**
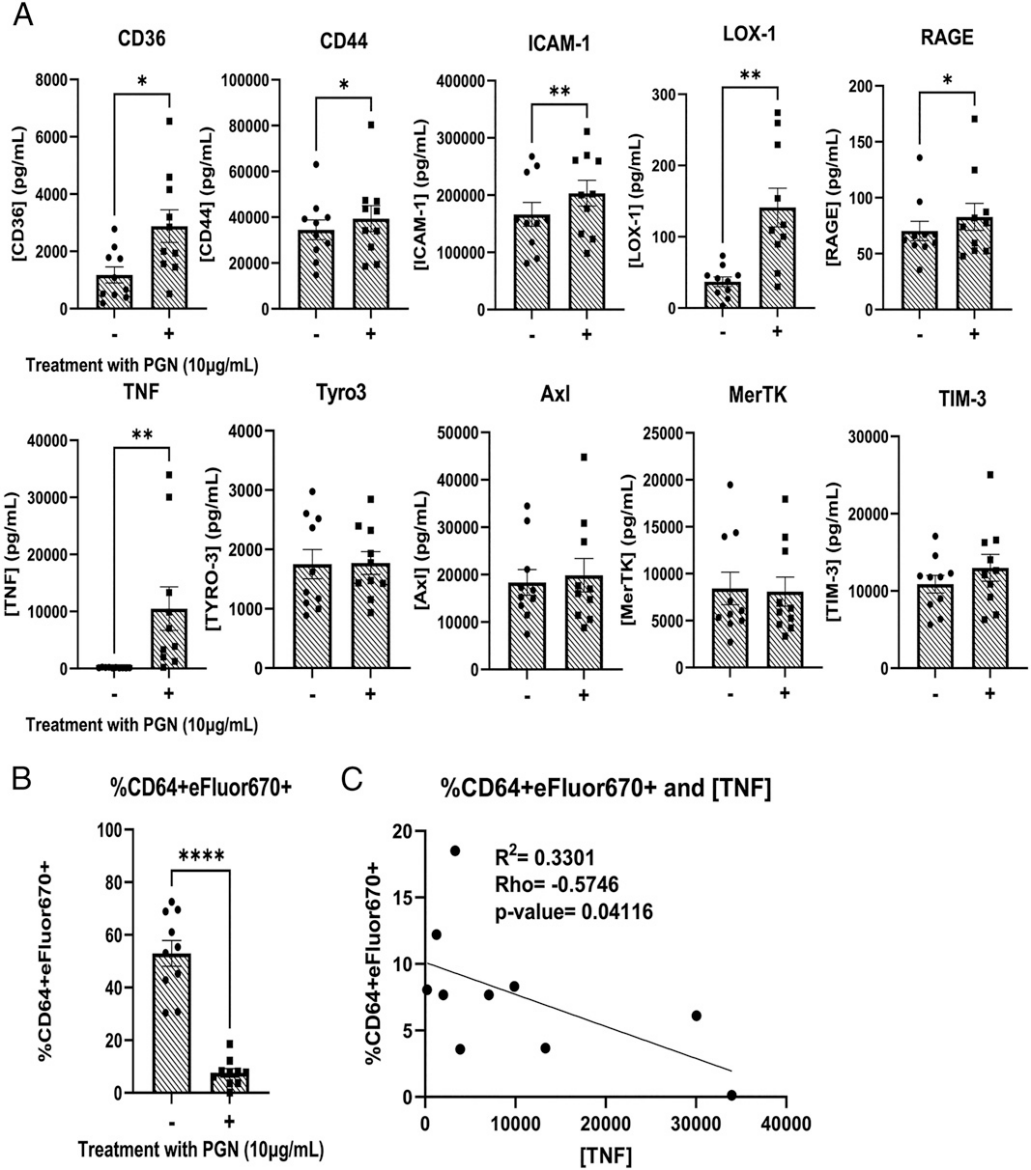
PGN-treated supernatants have increased levels of ADAM17 substrates. (**A**) Concentrations of soluble receptors and cytokines from efferocytosis supernatants that are known to be cleaved by ADAM17. (**B**) Percentage CD64^+^ eFluor 670^+^ efferocytosis from 10 donors. PGN-treated values were used for the correlation analysis. (**C**) Pearson correlation and linear regression analysis between percentage efferocytosis and concentration of TNF. PGN values are from (B). All analytes were measured from 24-h PGN-treated supernatants in the non-hihuS serum condition and analyzed by paired *t* tests. *p < 0.05, **p < 0.01, ****p < 0.0001.

Next, we investigated the cell surface expression of key efferocytosis signaling receptors in the presence of hiFBS, hihuS, and non-hihuS. We measured a total of 13 efferocytosis receptors and found significant changes in 6 of them: TYRO3, AXL, MERTK, integrin α_V_β_5_, TIM-3, and CD36 (Fig. 2A–2D). The remaining seven receptors showed low expression levels or were unchanged following PGN treatment (Supplemental Fig. 3A–3D). All of the significantly reduced cell surface receptors, except integrin α_V_β_5_, have been shown to be regulated via receptor cleavage (42). Next, we measured levels of multiple efferocytosis-associated molecules using a custom bead-based multiplex assay (Fig. 3). ADAMs are membrane-bound metalloproteases that act as sheddases to cleave various receptors from the cell membrane (43). In particular, ADAM17 is one of the well-studied ADAM family members and is known to cleave a multitude of receptors, including TNF (26), TYRO3 (44), AXL (45), MERTK (46), CD36 (47), TIM-3 (48), CD44 (49), ICAM-1 (50), LOX-1 (51), and RAGE (52). Of the 10 ADAM17-mediated receptors/cytokines we measured, 6 increased in PGN-treated supernatants; however, soluble forms of TYRO3, AXL, MERTK, and TIM-3 were unchanged (Fig. 3A, 3B). We observed an inverse correlation between concentration of TNF and efferocytosis (Fig. 3C, 3D); however, TNF blockade failed to prevent the PGN-induced defect (Supplemental Fig. 4). Overall, PGN reduced the cell surface expression of six key efferocytosis receptors on human M2-like MФ and increased multiple products of ADAM17 cleavage, suggesting involvement of membrane-bound proteases.

### Blockade of ADAM17 abolished TNF release but only modestly preserved efferocytic capacity and receptor expression

**FIGURE 4.**
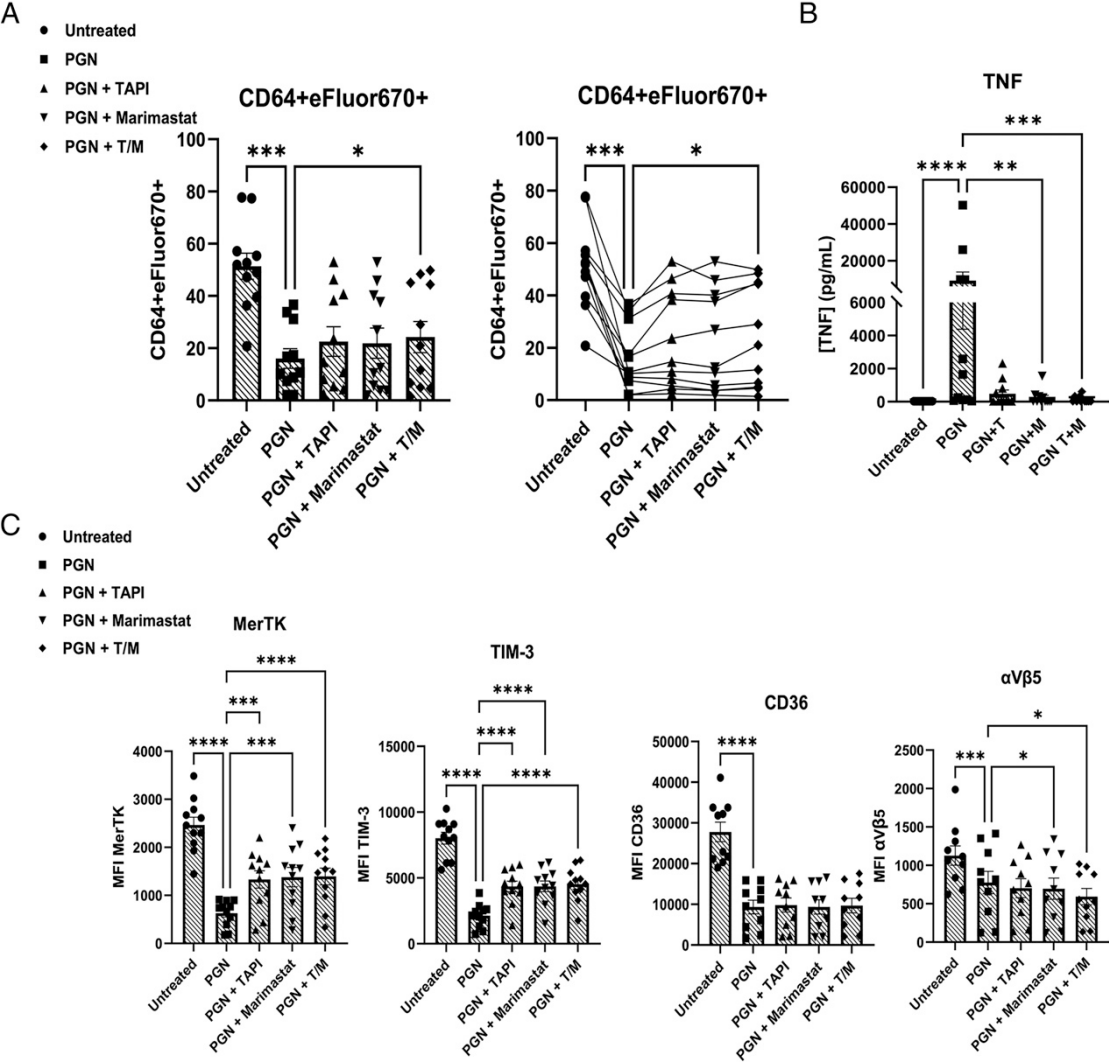
Pharmacological inhibition of ADAM17 partially restores surface receptor expression and efferocytic capacity. (**A**) Mean percentage efferocytosis (left) or an alternative display of the data showing the same donor tracked across each treatment group (right). (**B**) TNF concentrations from donor supernatants. (**C**) Level of receptor expression on cell surface (mean fluorescence intensity [MFI]). Data are from 11 independent donors after 16-h pretreatment with 10 µg/ml PGN in the presence of 10% non-hihuS serum alone or in combination with ADAM17 inhibitors. (A), (B), and (C) are paired data from the same donors. *p < 0.05, **p < 0.01, ***p < 0.001, ****p < 0.0001.

Regulation of ADAM17 activity is complex, based on its maturation and trafficking between the endoplasmic reticulum and Golgi and its subcellular localization in lysosomal and recycling compartments (53, 54). Using two small-molecule inhibitors, TAPI-0 and Marimastat, we evaluated if blocking ADAM17 could restore efferocytosis and receptor expression. Although TAPI-0 and Marimastat follow a similar mechanism of inhibition, by chelating zinc ions in the active site of ADAM17 and related proteases, TAPI-0 is not membrane permeable and inhibits proteases at the cell surface, whereas Marimastat can permeate cellular membranes (55). First, we titrated TAPI-0 (1, 30, 60 µM) and found no difference in efferocytosis among the three conditions (Supplemental Fig. 5). Next, we tested either inhibitor alone or in combination and observed partial increases in MERTK and TIM-3 in the presence of Marimastat or the Marimastat/TAPI-0 combination and a minor, though statistically significant, increase in efferocytosis (Fig. 4A, 4C). Following treatment with PGN, supernatants from cultures containing the ADAM17 inhibitor TAPI-0 alone or in combination with Marimastat showed substantially reduced TNF levels, indicating effective inhibition of enzyme activity (Fig. 4B).

## Discussion

Although *B. anthracis* produces toxins that enable the pathogen to replicate to high numbers (56), PGN is an important factor that contributes to late sepsis-like pathology (28, 29, 57). During sepsis and other forms of inflammation, there is an accumulation of uncleared apoptotic cells and host DAMPs, including nucleosomes (58, 59), suggesting potential defects in efferocytosis. We previously showed that *B. anthracis* edema toxin can inhibit efferocytosis by human M2-like MΦ (27). In the present study, we investigated the effect of PGN on human MФ efferocytosis. In this study, we report that PGN decreases efferocytosis in a human serum-dependent manner. We also provide limited evidence that MΦ recognition of PGN is independent of complement component C3, one of the most abundant and promiscuous serum opsonins. These findings agree with previous reports showing that other human immune cells generally require human serum opsonins for effective responses to polymeric PGN (60, 61). However, human MΦ did not require complement component C3 to respond to PGN, which differs from human PMNs (37). Further studies will be required to identify the key human serum opsonins responsible for enhanced recognition of *B. anthracis* PGN by human MΦ.

To investigate potential mechanisms leading to defective efferocytosis, we measured cell surface expression of key receptors that signal for apoptotic cell engulfment, including TAM (TYRO, AXL, MERTK), TIM (TIM-1, TIM-3), integrin (α_V_β_3_, α_V_β_5_), CD300 (CD300b, CD300f), and stabilin (STABILIN-1, STABILIN-2) families, as well as the thrombospondin receptor, CD36. Although the transmembrane receptor BAI-1 is considered to be an efferocytosis signaling receptor in mice (62), a recent publication failed to find expression of this receptor at the protein or RNA level in human MФ (63); therefore, we excluded this receptor from our screening. In total, cell surface expression of six receptors was significantly reduced following exposure to *B. anthracis* PGN for 24 h (TYRO3, AXL, MERTK, TIM-3, α_V_β_5_, and CD36), suggesting that downregulation of these receptors might be responsible for PGN-mediated defects in our model.

One known mechanism of regulation, common to multiple efferocytosis receptors, is enzyme-mediated shedding. In humans, ADAM17 is known to cleave multiple efferocytosis receptors, resulting in their soluble forms (22). Cleavage of receptors can prevent efferocytosis by reducing the cell surface expression of the receptor, and soluble forms act as decoy receptors to bind phosphatidylserine sites and inhibit recognition of apoptotic cells by other MΦ. Previous work by Michlewska et al. has shown that LPS inhibits efferocytosis in a TNF-dependent mechanism in unpolarized human MΦ (64) and that IL-10 and TNF show opposing secretion profiles. In contrast, our work demonstrated that both IL-10 and TNF increase following treatment with PGN. We further showed that TNF was not responsible for *B. anthracis* PGN-induced defective efferocytosis by human M2-like MΦ. Our data showed that both protease inhibition, which prevented TNF release, and TNF neutralization failed to substantially restore efferocytosis. Michlewska et al. pretreated nonpolarized MΦ with LPS for an extended period of time (120 h), resulting in much higher TNF levels; thus, differences between the studies may be attributable to differing MΦ activation states and differences in TNF levels. Consistent with PGN-induced sheddase activity, we observed significant increases in the soluble forms of CD36, as well as ICAM-1, CD44, LOX-1, and RAGE, key efferocytosis receptors that were reported to be cleaved by ADAM17.

To block ADAM17, we used two small-molecule inhibitors, TAPI-0 and Marimastat, and saw partial restoration of efferocytosis as well as a partial increase in MERTK and TIM-3 on the cell surface; however, neither returned to baseline levels. In addition to inhibiting ADAM17, TAPI-0 and Marimastat also inhibit other related proteases, including MMPs (65, 66). Thus, the modestly increased expression of MERTK and TIM-3 in the presence of these inhibitors is consistent with cleavage of these receptors by a range of possible proteases in response to PGN. Interestingly, we did not observe changes in the levels of soluble TYRO3, AXL, MERTK, or TIM-3, suggesting that *B. anthracis* PGN induces selective substrate cleavage and/or alternative regulation beyond cell surface expression. Work by Maretzky et al. has shown that ADAM17 can cleave different substrates when in the presence of rhomboid proteins 1 or 2 (iRhom1, iRhom2), demonstrating that when complexed together, iRhom proteins can modify ADAM17 substrate specificity (67, 68). To the best of our knowledge, substrate specificity as a mechanism of receptor regulation has yet to be shown for the TYRO, AXL, MERTK (TAM) family. Of note, the TAM family was demonstrated to be regulated through intracellular cleavage at their membrane-proximal region by γ-secretase. Merilahti et al. demonstrated that intracellular TAM family members could interact with cytosolic signaling intermediates and also localize to the nucleus (69). In addition, it has been noted in human MΦ from patients with systemic lupus erythematosus that the level of soluble receptor and membrane-bound forms of MERTK do not correlate (70). This suggests nuanced regulation and could potentially explain our result of reduced cell surface expression in PGN-treated MΦ yet an equal level of soluble receptor in PGN-treated and untreated MΦ supernatants. Overall, this study suggests the existence of additional mechanisms for downregulation of these receptors and for PGN-mediated impairment of efferocytosis, some of which may be transcriptional or post-translational.

Although PGNs are present in both Gram^+^ and Gram^–^ bacteria, the PGN layer is much thicker in Gram^+^ species, and therefore the pathological burden of PGN is expected to be higher during Gram^+^ infections such as anthrax. Furthermore, the structural heterogeneity among bacterial PGNs, such as variations in stem peptides and post-synthetic modifications of the glycan strands (71), could lead to diverse biologically active breakdown products that in turn alter downstream signaling and functional outcomes (32, 71, 72, 73). Further studies are required to determine whether other bacterial PGNs can inhibit the capacity of human MΦ to engulf apoptotic cells.

In summary, we report that PGN from *B. anthracis* inhibits efferocytosis by human M2-like MФ and that human serum is important for eliciting maximal response. We also demonstrate that PGN-mediated defects are partially due to ADAM17-mediated proteolysis of MERTK and TIM-3 receptors. Nevertheless, ADAM17 inhibition only partially restores PGN-induced efferocytic impairment; thus, metalloprotease-independent mechanisms for regulating efferocytic receptors must also occur. Overall, these data show that bacterial factors can affect the capacity of MФ to efferocytose and provide a new, to our knowledge, explanation for the accumulation of uncleared apoptotic cells in the context of late-stage anthrax infection.

## Supplementary Material

Supplemental Material (PDF)
