# Supplementary material for: Peptidoglycan from Bacillus anthracis Inhibits Human Macrophage Efferocytosis in Part by Reducing Cell Surface Expression of MERTK and TIM-3
Source: Immunohorizons. 2024 Mar 22;8(3):269–80. doi: 10.4049/immunohorizons.2300109 (PMC10985058; doi:10.4049/immunohorizons.2300109)
Supplement: Supplemental Material (PDF) [file IH_2300109_Supplemental_1.pdf]

SUPPLEMENTAL FIGURES

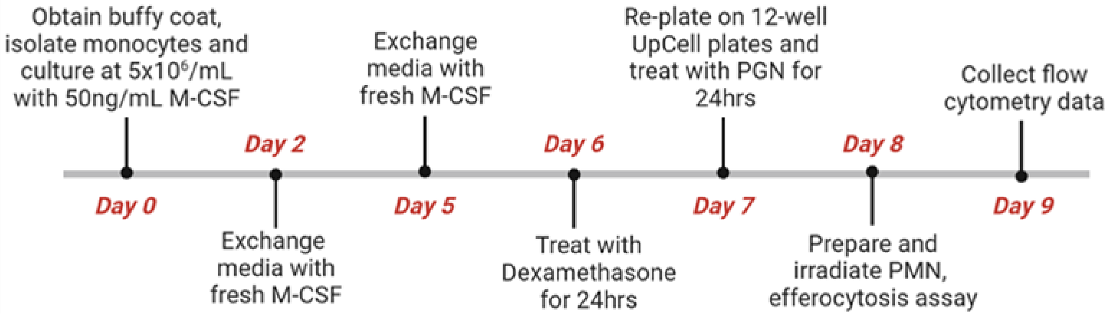

Supplemental Figure 1: Overview of experimental setup. On Day 0 MΦ were isolated from buffy coat donors, adhered to plates for 1h, and cultured with M-CSF. On Day 2 and Day 5 half of the media was replaced with fresh complete media supplemented with 2x M-CSF to ensure differentiation. MΦ were polarized with Dexamethasone on Day 6, and 24hrs later, were re-plated onto 12-well UpCell plates and stimulated with various treatments. On Day 8 PMN were isolated, irradiated, co-cultured with MΦ for 1hr and subsequently stained for flow cytometry. PGN-treated supernatant was removed prior to PMN co-culture. On Day 9 samples were stained for flow cytometry, and data were collected (LSRII) and analyzed (FlowJo). Figure was created using Biorender, Template (9 Segments, Horizonal), by BioRender.com (2023). Retrieved from https://app.biorender.com/biorender-templates.

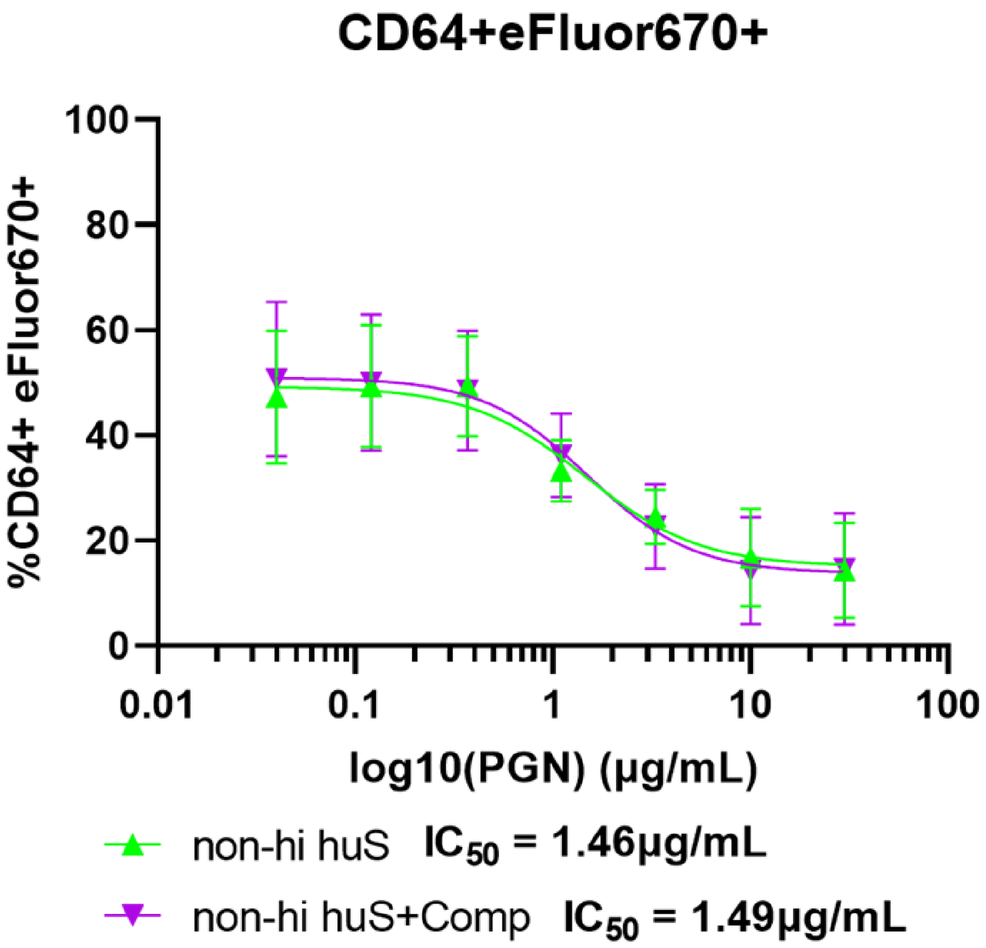

Supplemental Figure 2: Dose response of PGN on MΦ efferocytosis from non-hi huS or non-hi huS+ Compstatin conditions from 3 independent donors (30µg/mL-0.04µg/mL). Data was log(10)-transformed and graphed using a non-linear fit model in Prism. Dose-response curve was used to calculate IC_{50}.

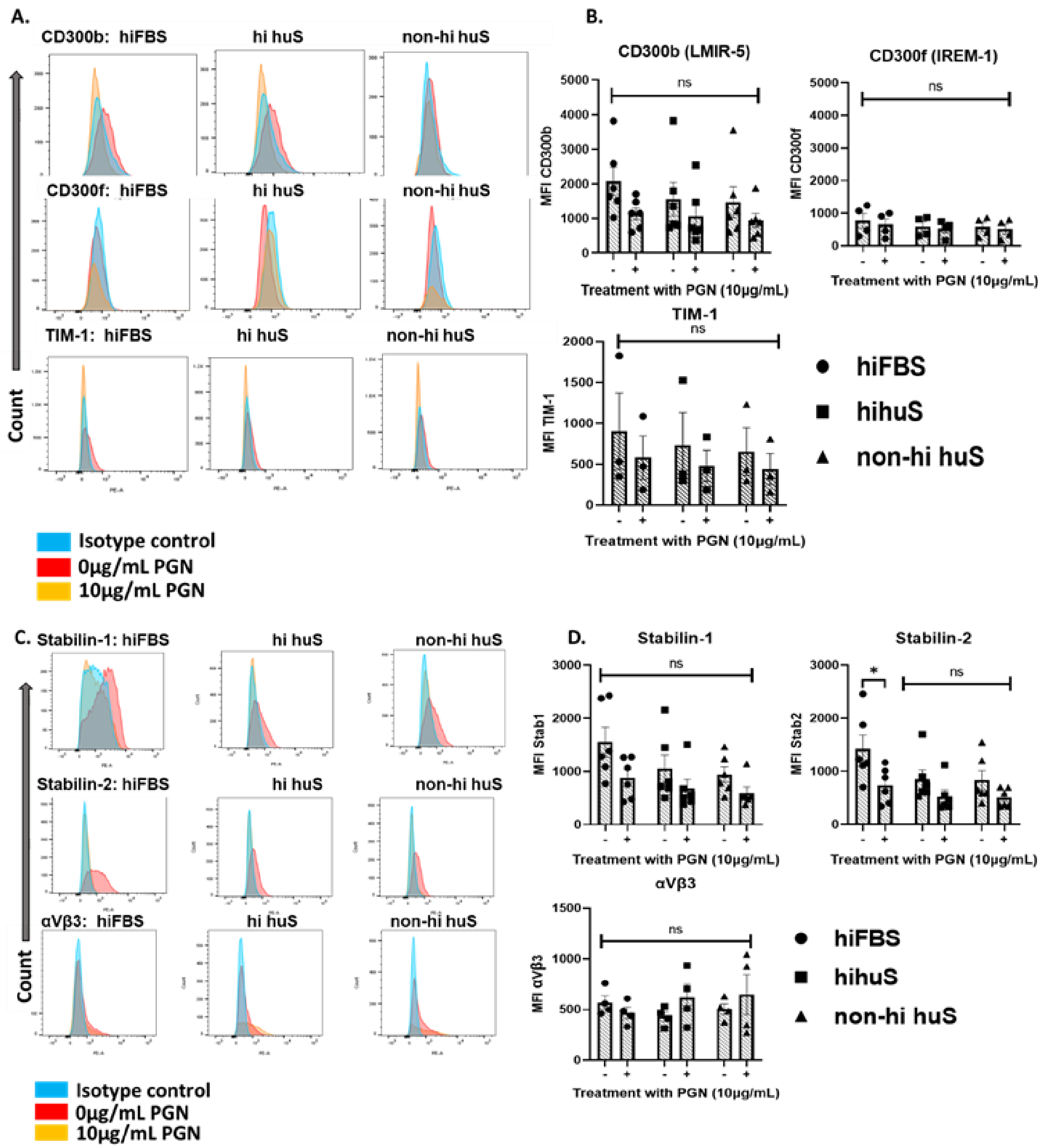

Supplemental Figure 3: Efferocytosis receptors not affected by PGN treatment in non-hi huS in human M2-like macrophages. **(A, C)** Representative histograms of receptor expression**. (B, D)** MFI of surface efferocytosis receptors from the indicated serum conditions from ≥3 independent donors treated with 10µg/mL PGN for 24hrs and analyzed by two-way ANOVA with Sidak's multiple comparison.

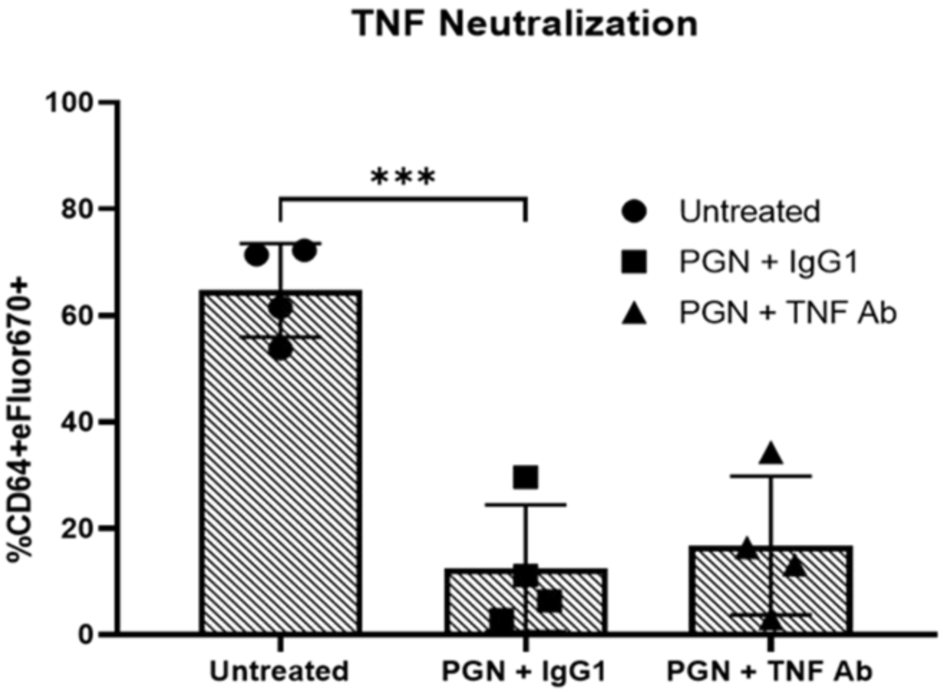

Supplemental Figure 4: TNF neutralization does not rescue PGN-induced defects in efferocytosis. Percentage of CD64+ MΦ containing apoptotic eFluor670-labeled PMN treated with 6µg/mL of either an IgG isotype control (β-Galactosidase, InvivoGen Cat#: bgal-mab1) or human anti-TNF (IgG1, InvivoGen, Cat#: htnf-mab1) antibody in the presence of 10ug/mL PGN for 24 hrs. 4 independent donors analyzed by one-way ANOVA with Dunnet's multiple comparison.

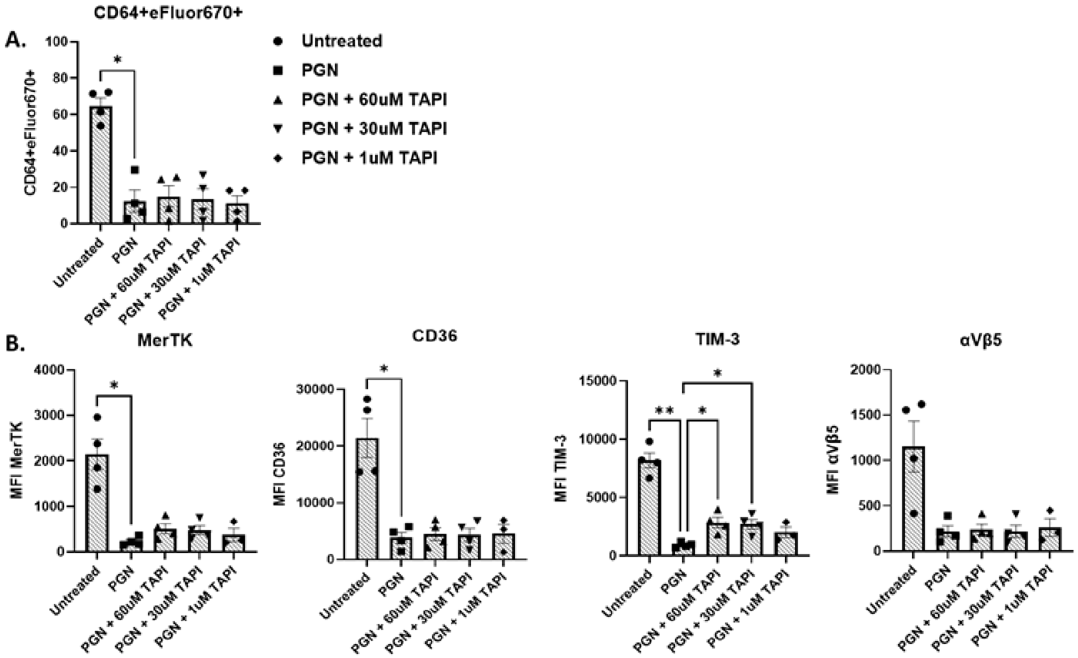

Supplemental Figure 5: A) Percentage of CD64+ MΦ containing apoptotic eFluor670-labeled PMN from the non-hi huS condition in the presence of absence of 10ug/mL PGN and various doses of TAPI-0 (60, 30, 1µM). **B)** MFI of efferocytosis receptors from the same donors in Figure 5A. Data is from ≥3 independent and was analyzed using a mixed effects model with Sidak's multiple comparison due to a missing data point for the 1µM TAPI condition.
